# Bisphosphonates and Risk of Cardiovascular Events: A Meta-Analysis

**DOI:** 10.1371/journal.pone.0122646

**Published:** 2015-04-17

**Authors:** Dae Hyun Kim, James R. Rogers, Lisa A. Fulchino, Caroline A. Kim, Daniel H. Solomon, Seoyoung C. Kim

**Affiliations:** 1 Division of Pharmacoepidemiology and Pharmacoeconomics, Department of Medicine, Brigham and Women’s Hospital, Harvard Medical School, Boston, Massachusetts, United States of America; 2 Division of Rheumatology, Immunology, and Allergy, Department of Medicine, Brigham and Women’s Hospital, Harvard Medical School, Boston, Massachusetts, United States of America; 3 Division of Gerontology, Department of Medicine, Beth Israel Deaconess Medical Center, Harvard Medical School, Boston, Massachusetts, United States of America; University of Bologna, ITALY

## Abstract

**Background and Objectives:**

Some evidence suggests that bisphosphonates may reduce atherosclerosis, while concerns have been raised about atrial fibrillation. We conducted a meta-analysis to determine the effects of bisphosphonates on total adverse cardiovascular (CV) events, atrial fibrillation, myocardial infarction (MI), stroke, and CV death in adults with or at risk for low bone mass.

**Methods:**

A systematic search of MEDLINE and EMBASE through July 2014 identified 58 randomized controlled trials with longer than 6 months in duration that reported CV events. Absolute risks and the Mantel-Haenszel fixed-effects odds ratios (ORs) and 95% confidence intervals (CIs) of total CV events, atrial fibrillation, MI, stroke, and CV death were estimated. Subgroup analyses by follow-up duration, population characteristics, bisphosphonate types, and route were performed.

**Results:**

Absolute risks over 25–36 months in bisphosphonate-treated versus control patients were 6.5% versus 6.2% for total CV events; 1.4% versus 1.5% for atrial fibrillation; 1.0% versus 1.2% for MI; 1.6% versus 1.9% for stroke; and 1.5% versus 1.4% for CV death. Bisphosphonate treatment up to 36 months did not have any significant effects on total CV events (14 trials; ORs [95% CI]: 0.98 [0.84–1.14]; *I^2^* = 0.0%), atrial fibrillation (41 trials; 1.08 [0.92–1.25]; *I^2^* = 0.0%), MI (10 trials; 0.96 [0.69–1.34]; *I^2^* = 0.0%), stroke (10 trials; 0.99 [0.82–1.19]; *I^2^* = 5.8%), and CV death (14 trials; 0.88 [0.72–1.07]; *I^2^* = 0.0%) with little between-study heterogeneity. The risk of atrial fibrillation appears to be modestly elevated for zoledronic acid (6 trials; 1.24 [0.96–1.61]; *I^2^* = 0.0%), not for oral bisphosphonates (26 trials; 1.02 [0.83–1.24]; *I^2^* = 0.0%). The CV effects did not vary by subgroups or study quality.

**Conclusions:**

Bisphosphonates do not have beneficial or harmful effects on atherosclerotic CV events, but zoledronic acid may modestly increase the risk of atrial fibrillation. Given the large reduction in fractures with bisphosphonates, changes in osteoporosis treatment decision due to CV risk are not justified.

## Introduction

Over 30 million older adults in the United States have low bone mass.[1] These individuals have greater burden of vascular calcification and rapidly progressive atherosclerosis compared with those with normal bone mass.[2–5] Every 1 standard deviation decrease in bone mineral density is associated with 1.3 to 2.3-fold increase in cardiovascular (CV) mortality.[6–8] In fact, emerging evidence indicates that vascular calcification is an actively regulated process that shares several biological mechanisms with bone mineralization.[9–11] In vitro and in vivo studies have demonstrated the potential of vascular cells to undergo osteoblastic differentiation.[12,13] Likewise, osteoclast-like cells have been found in the human calcified atherosclerotic lesions.[14] Bone matrix proteins and regulatory factors appear to regulate vascular calcification.[11]

Understanding the biological similarities between vascular calcification and bone mineralization has led to the idea that bisphosphonates may influence vascular calcification. In addition to inhibiting bone resorption, evidence suggests that bisphosphonates may inhibit atherosclerosis and vascular calcification. In several small-scale clinical trials, etidronate modestly improved surrogate endpoints of atherosclerosis, such as carotid intima media thickness,[15] coronary artery calcium,[16] and aortic calcification.[17] However, bisphosphonate trials designed to study clinical CV endpoints are currently lacking. Epidemiologic studies found a lower risk of myocardial infarction (MI) or stroke among bisphosphonate users compared with non-users.[18–20] While such epidemiologic evidence suggests that bisphosphonates may protect against atherosclerotic CV events,[21,22] healthy user bias cannot be excluded. Moreover, a possible increase in atrial fibrillation associated with bisphosphonates[23,24] may counteract the potential benefits on atherosclerotic CV disease.

In the absence of any bisphosphonate trial sufficiently powered to examine clinical CV events, a meta-analysis of previously conducted trials would be a useful approach to explore potential CV effects of bisphosphonates. Prior reviews have focused on atrial fibrillation[25–34] or a single bisphosphonate agent.[31–33] We conducted a meta-analysis of randomized controlled trials (RCTs) to determine the effects of bisphosphonates versus placebo or no treatment control on total CV events, atrial fibrillation, MI, stroke, and CV mortality in adults with or at risk for low bone mass.

## Methods

### Data Sources and Systematic Search

This meta-analysis was conducted in accordance with PRISMA guidelines (http://www.equator-network.org/reporting-guidelines/prisma/; review protocol available from authors upon request). We performed a systematic search of MEDLINE and EMBASE, with no language restriction, from inception through July 28, 2014, to identify RCTs of bisphosphonates (see Text 1 in S1 File for systematic search strategy). Because adverse events were incompletely indexed in MEDLINE and EMBASE,[35] we did not use any keywords for CV events in our search. Instead, we manually looked for information on adverse CV events (total adverse CV events, atrial fibrillation, MI, stroke, and CV mortality) in the full text. We searched references in published reviews[25–34] to obtain additional CV event data that were unavailable in the original publications. We included 2 pooled analyses by Lewiecki et al. (4 trials of ibandronate)[32] and Karam et al. (6 trials of risedronate).[33] We also contacted the authors of publications within the recent 10 years that included at least 1000 participants, but our attempt did not yield any additional information. Because this was a meta-analysis, ethics approval was not required.

### Study Selection

Trained research assistants (JRR and LAF) independently evaluated all identified publications for their eligibility and any disagreements were resolved through discussion with investigators (DHK and SCK). A publication was considered eligible if it originated from RCTs that compared oral or intravenous bisphosphonate versus placebo or no treatment control in adults. We excluded a publication for the following reasons: 1) not RCT of oral or intravenous bisphosphonate versus no bisphosphonate (e.g. no randomization, cross-over design, open-label extension study, or lack of control group); 2) the follow-up duration of 6 months or shorter; 3) conducted in specific populations that might have a different risk of CV events (e.g. patients with cancer, transplant, or human immunodeficiency virus infection, or children); 4) study protocols, commentaries, reviews, or non-human studies; 5) no data on total CV events, atrial fibrillation, MI, stroke, and CV mortality.

### Data Extraction and Assessment of Study Quality

Using a standardized form, 3 reviewers (JRR, LAF, and DHK) extracted the following information from each study: bisphosphonate (dose, frequency, and route), sample size, mean age (years), female (%), mean weight (kg), mean body mass index (kg/m^2^), characteristics of study population (osteoporosis or other at-risk conditions for osteoporosis, such as osteopenia, steroid use, or chronic inflammatory disease), follow-up duration (months), and number of CV events, including serious atrial fibrillation events, defined as death, disability, hospitalization, or requiring interventions related to atrial fibrillation. Three investigators (DHK, CAK, and SCK) confirmed the accuracy of data collection and assessed the study quality as adequate or inadequate, according to the following standards: 1) generation of random sequence; 2) concealment of randomization; 3) blinding of participants and study personnel; 4) blinding of outcome assessors; 5) loss to follow-up (adequate if loss to follow-up was less than 20%); 6) completeness of CV event reporting (complete if the number of atrial fibrillation, MI, stroke, and CV death was reported); 7) ascertainment of CV events (adequate if CV events were adjudicated based on medical records). When there was insufficient information, it was considered inadequate.

### Data Synthesis and Analysis

For each of CV events, we combined individual study results to calculate the pooled odds ratio (OR) and 95% confidence interval (CI) using the Mantel-Haenszel fixed-effects method without zero-cell correction. This method is the least biased method in analyzing sparse events when there is large imbalance in the treatment group size.[36] Between-study heterogeneity was assessed using the Cochran’s *Q* statistic and *I* ^*2*^ statistic.[37] Pre-specified subgroup meta-analyses and meta-regression were performed to evaluate whether the CV effects of bisphosphonates differed by follow-up duration (≤12, 13–24, 25–36, >36 months), age (<60, 60–69, ≥70 years), sex (female, male, or both), population characteristics (osteoporosis or at-risk conditions for osteoporosis), bisphosphonate types (alendronate, ibandronate, risedronate, zoledronic acid, or others), and route (oral or intravenous). We summarized the risk of CV events by bisphosphonate dose, but did not perform a formal dose-response analysis due to limited numbers of trials that reported each outcome.

As sensitivity analyses, we assessed the influence of study quality standards on the pooled OR. We also examined whether the pooled ORs were due to any single influential study by repeating meta-analyses after excluding 1 study at a time. Since CV events were not reported in all trials, we investigated the possibility of publication bias using the Begg adjusted rank correlation test,[38] the Egger regression test,[39] and the trim-and-fill method (Stata METATRIM command). All analyses were performed in Stata version 11 (StataCorp, College Station, TX). A 2-sided p-value <0.05 was considered statistically significant.

## Results

### Characteristics of Included Studies

**Fig 1:**
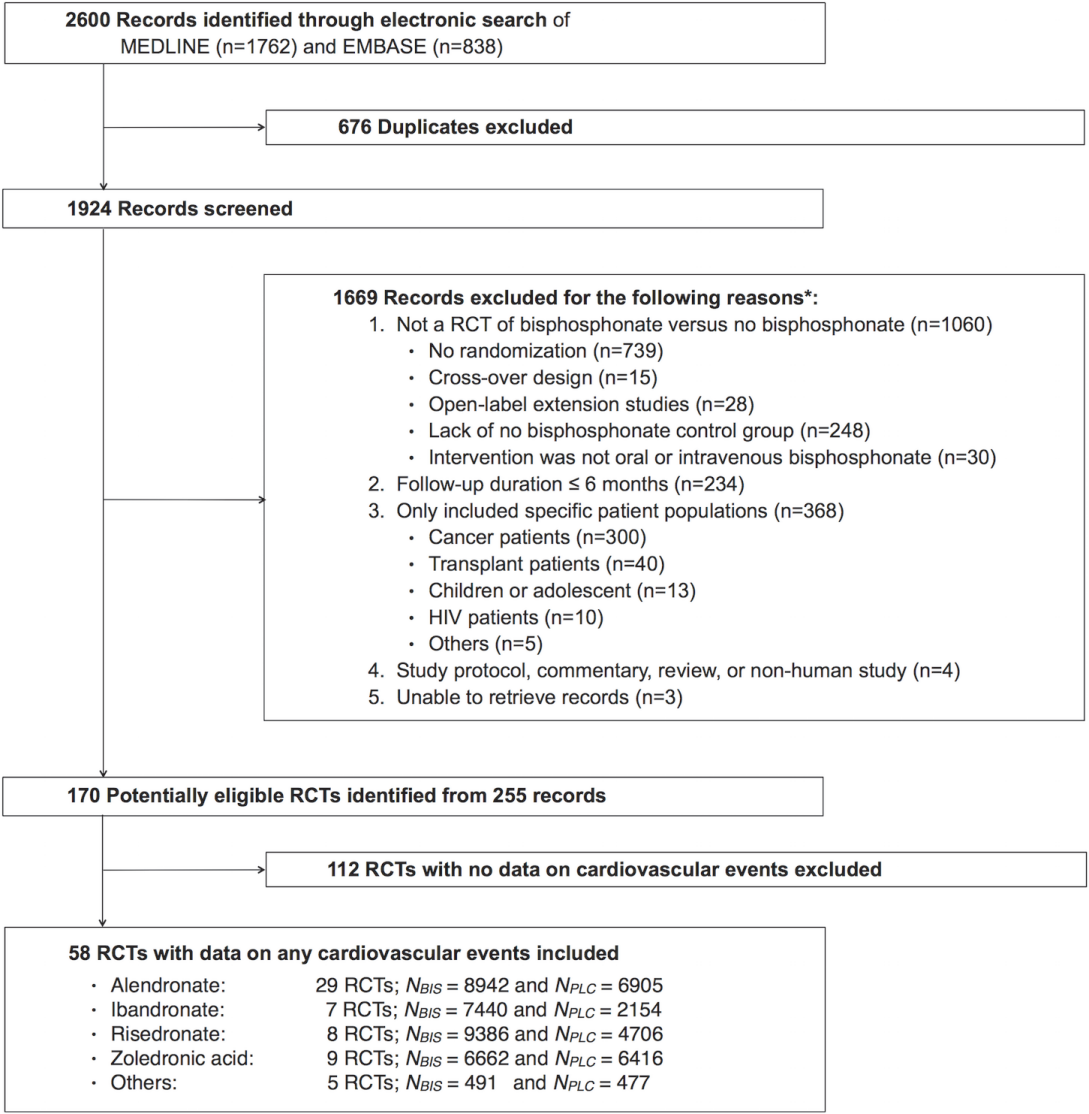
Selection of Randomized Controlled Trials of Bisphosphonates for Meta-Analysis. Abbreviations: BIS, bisphosphonate; CTR, control; HIV, human immunodeficiency virus; RCT, randomized controlled trial

A total of 58 RCTs reported at least 1 CV outcome (Fig 1): total adverse CV events (14 trials), atrial fibrillation (41 trials), MI (10 trials), stroke (10 trials), and CV mortality (14 trials). Compared with 112 trials without CV event data, 58 trials with CV event data had larger sample size (median: 158 versus 37 in bisphosphonate group and 92 versus 35 in control group), older participants (mean age: 63.0 versus 58.8 years), and longer follow-up time than trials without CV event data (median: 24 versus 12 months) (Table 1 in S1 File). Most trials included adults who were generally healthy and without multiple comorbidities (Table 2 in S1 File).

### Assessment of Study Quality

The quality of the 58 included trials was summarized in Fig 1 in S1 File (see Table 3 in S1 File for individual study quality). The number of trials that satisfied each quality standard varied widely: generation of random sequence (28%), concealment of allocation (16%), blinding of participants and personnel (93%), blinding of outcome assessors (95%), adequacy of follow-up (74%), completeness of CV event reporting (5%), and ascertainment of CV events (5%).

### Bisphosphonates and Total Adverse CV Events

**Fig 2:**
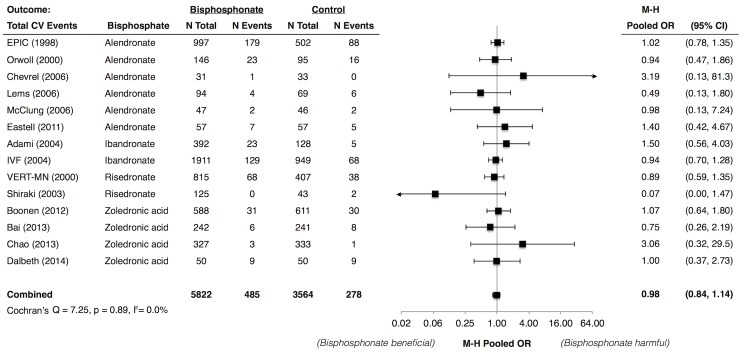
Meta-Analysis of Total Adverse Cardiovascular Events Associated with Use of Bisphosphonates. Abbreviations: CI, confidence interval; CV, cardiovascular; EPIC, Early Postmenopausal Intervention Cohort study; IVF, IntraVenous Fracture study; M-H, Mantel Haenszel; OR, odds ratio; VERT-MN, Vertebral Efficacy with Risedronate Therapy Multinational Study

Total adverse CV event data were available in 14 trials that included 5822 bisphosphonate-treated and 3564 control patients. The absolute risk was 6.5% in bisphosphonate-treated patients versus 6.2% in control patients over 25–36 months. The Mantel-Haenszel pooled OR (95% CI) was 0.98 (0.84, 1.14) with little between-study heterogeneity (*I* ^*2*^ = 0.0%) (Fig 2). The pooled ORs did not differ across subgroups defined by the follow-up duration, mean age, sex, population characteristics, bisphosphonate types, and administration route (Fig 2 in S1 File).

### Bisphosphonates and Atrial Fibrillation

**Fig 3:**
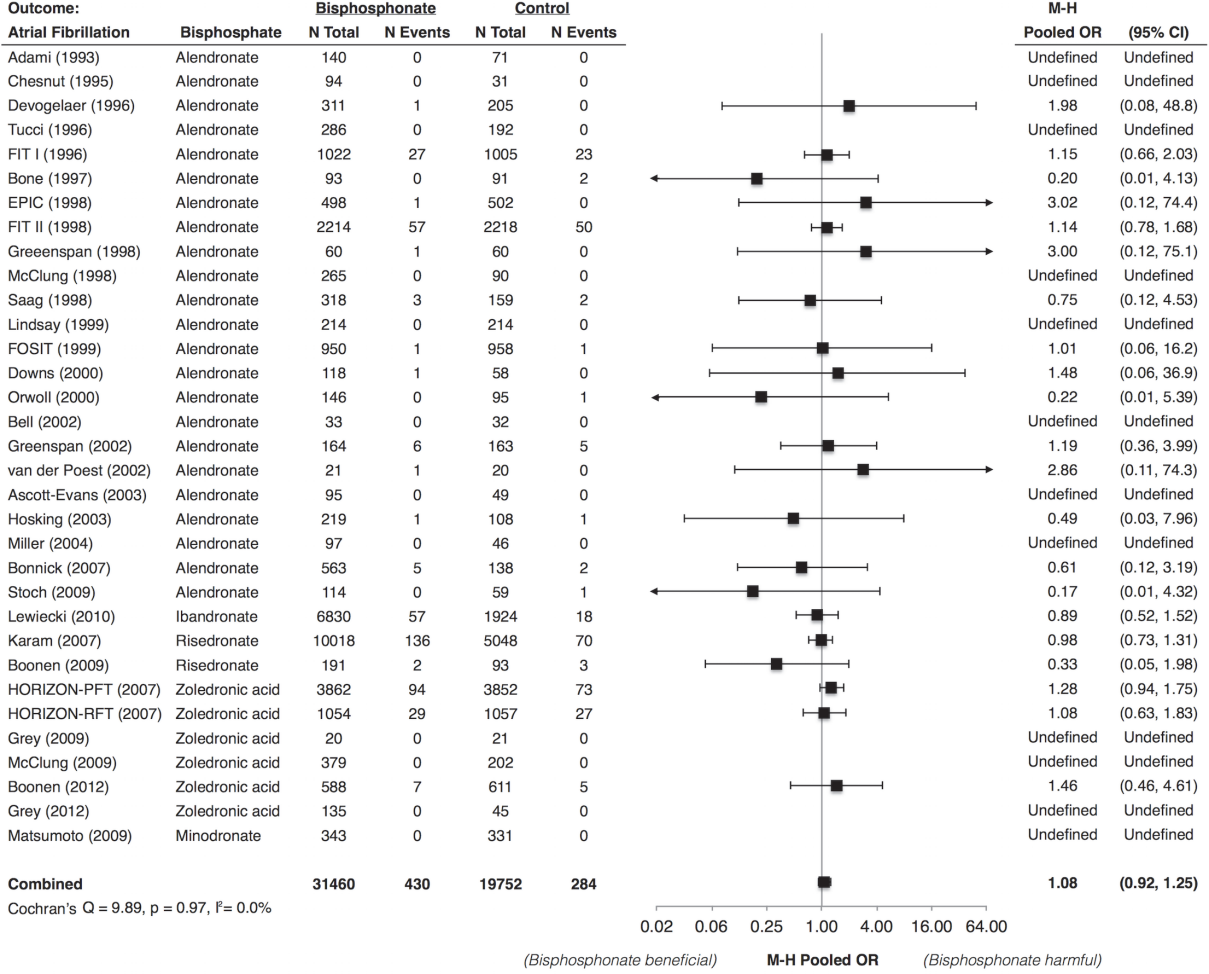
Meta-Analysis of Atrial Fibrillation Associated with Use of Bisphosphonates. * Abbreviations: CI, confidence interval; EPIC, Early Postmenopausal Intervention Cohort study; FIT, Fracture Intervention Trial; FOSIT, Fosamax International Trial; HORIZON-PFT, the Health Outcomes and Reduced Incidence with Zoledronic Acid Once Yearly Pivotal Fracture Trial; HORIZON-RFT, the Health Outcomes and Reduced Incidence with Zoledronic Acid Once Yearly Recurrent Fracture Trial; M-H, Mantel Haenszel; OR, odds ratio. * Lewiecki (2010) et al. included data from 4 trials of ibandronate. Karam (2007) et al. included data from 6 trials of risedronate. Individual study data were not available.

Data on atrial fibrillation were available in 41 trials that included 31460 bisphosphonate-treated and 19752 control patients. The absolute risk was 1.4% in bisphosphonate-treated patients and 1.5% in control patients over 25–36 months. The Mantel-Haenszel pooled OR (95% CI) was 1.08 (0.92, 1.25) with little between-study heterogeneity (*I* ^*2*^ = 0.0%) (Fig 3). There was no statistically significant variation in the pooled ORs across pre-defined subgroups (Fig 3 in S1 File). However, the risk seemed modestly elevated for zoledronic acid (pooled OR: 1.24; 95% CI: 0.96, 1.61) (Fig 3 in S1 File).

When the analysis was restricted to serious atrial fibrillation events available in phase 3 trials,[23,24,32,33,40] there was a modestly increased risk (pooled OR: 1.41; 95% CI: 1.10, 1.81) with large between-study heterogeneity (*I* ^*2*^ = 78.4%). The elevated risk and heterogeneity were mainly driven by a single study of zoledronic acid[23]: the pooled OR (95% CI) after excluding this study was 1.19 (0.90, 1.58) with little between-study heterogeneity (*I* ^*2*^ = 0.0%).

### Bisphosphonates and MI, Stroke, and CV Death

**Fig 4:**
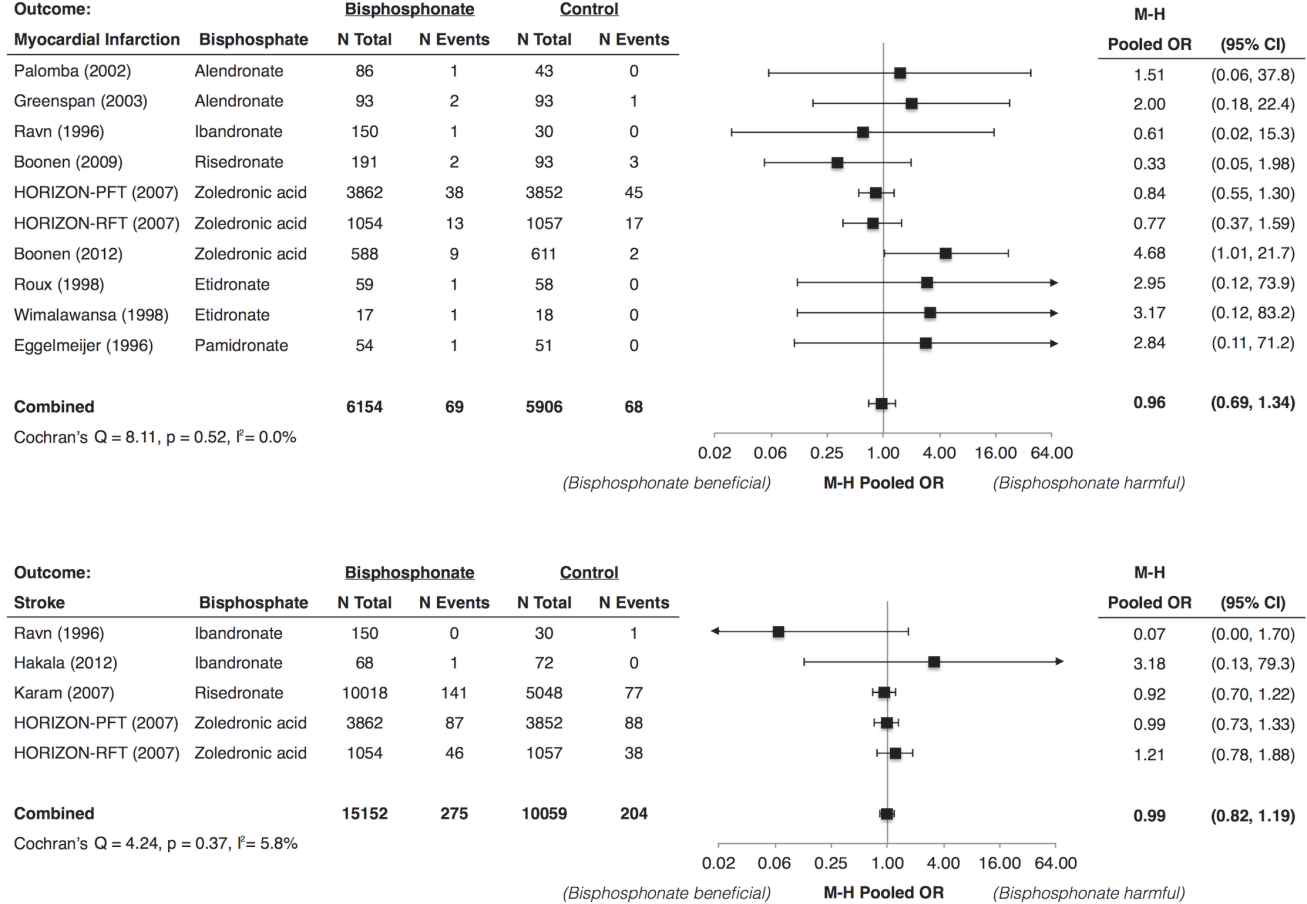
Meta-Analysis of Myocardial Infarction and Stroke with Use of Bisphosphonates. * Abbreviations: CI, confidence interval; HORIZON-PFT, the Health Outcomes and Reduced Incidence with Zoledronic Acid Once Yearly Pivotal Fracture Trial; HORIZON-RFT, the Health Outcomes and Reduced Incidence with Zoledronic Acid Once Yearly Recurrent Fracture Trial; M-H, Mantel Haenszel; OR, odds ratio. * Karam (2007) et al. included data from 6 trials of risedronate. Individual study data were not available.

**Fig 5:**
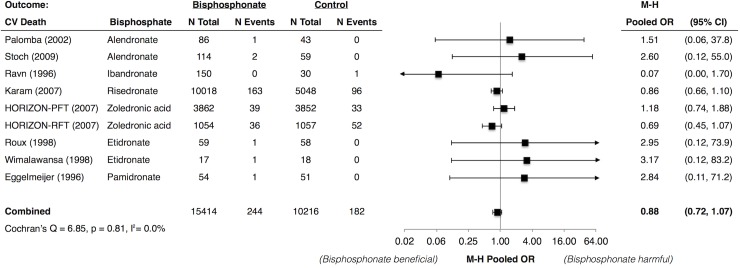
Meta-Analysis of Cardiovascular Mortality with Use of Bisphosphonates. * Abbreviations: CI, confidence interval; CV, cardiovascular; HORIZON-PFT, the Health Outcomes and Reduced Incidence with Zoledronic Acid Once Yearly Pivotal Fracture Trial; HORIZON-RFT, the Health Outcomes and Reduced Incidence with Zoledronic Acid Once Yearly Recurrent Fracture Trial; M-H, Mantel Haenszel; OR, odds ratio. * Karam (2007) et al. included data from 6 trials of risedronate. Individual study data were not available.

The risks of MI, stroke, and CV death over 25–36 months in bisphosphonate-treated patients and control patients were low (MI: 1.0% versus 1.2%; stroke: 1.6% versus 1.9%; and CV death: 1.5% versus 1.4%) and similar between the treated and placebo patients (Figs 4 and 5). There was no statistically significant difference in the pooled ORs across the pre-defined subgroups (Figs 4, 5, and 6 in S1 File).

### Sensitivity Analyses

There was no indication of dose-response relationship between bisphosphonate dose and each CV event (Table 4 in S1 File). The quality standards had little effects on the pooled ORs (Table 5 in S1 File). Except serious atrial fibrillation events, our results changed minimally when we re-estimated the pooled ORs after excluding 1 trial at a time. Finally, we did not find any evidence of publication bias by the Begg adjusted rank correlation test and the Egger regression test for all 5 outcomes (data not shown). The trim-and-fill method made little difference in the pooled OR (95% CI): 0.98 (0.83, 1.14) for total CV events; 1.08 (0.93, 1.26) for atrial fibrillation; 0.82 (0.59, 1.15) for MI; 0.99 (0.82, 1.19) for stroke; and 0.87 (0.72, 1.06) for CV death. These results suggest that selective reporting of CV events among the trials was unlikely.

## Discussion

Our meta-analysis of 58 RCTs did not find any indication that treatment with commonly prescribed bisphosphonates (alendronate, ibandronate, risedronate, and zoledronic acid) up to 36 months or longer would have any clinically important effects on atherosclerotic CV events among individuals with or at risk for low bone mass. The risk of atrial fibrillation appears to be modestly elevated for intravenous zoledronic acid, although the evidence was inconclusive. These results contradict the findings from previous observational studies[18–20] and clinical trials of surrogate endpoints (e.g. carotid intima media thickness,[15,41] coronary artery calcium,[16] and aortic calcification[17,42]) that suggest possible benefits of bisphosphonates on atherosclerotic CV events.

Potential mechanisms by which bisphosphonates may reduce atherosclerotic CV events include inducing macrophage apoptosis, preventing foam cell formation, lowering cholesterol levels by inhibiting mevalonate pathway, and anti-inflammatory effect.[9,10,21,22] Bisphosphonate prevented development or decreased the extent of atherosclerosis in animal models.[43–45] In humans, etidronate has been studied in several trials as an inhibitor of vascular calcification. It decreased carotid intima media thickness (by 0.038 mm),[15] coronary artery calcium score (by 372 mm^3^),[16] and aortic calcification (by 14–15%)[17] in high-risk patients over 9–12 months. Such reduction in carotid intima media thickness was comparable to or greater than the effects observed from some statins: pitavastatin decreased carotid intima media thickness by 0.024 mm/year in patients with known atherosclerosis,[46] while rosuvastatin decreased carotid intima media thickness by 0.0014 mm/year among low-risk individuals.[47] The evidence on other bisphosphonates is mixed. Alendronate reduced carotid intima media thickness (by 0.025 mm)[41] in hemodialysis patients, but not in patients with chronic kidney disease.[48] While a 36-month ibandronate treatment did not alter the progression of aortic calcification,[49] risedronate slowed progression in 12 months.[42] Despite the beneficial effects on surrogate endpoints, we found no evidence of reduction in atherosclerotic CV events with bisphosphonates (as opposed to statins). Some evidence suggests that most benefits of statins result from plaque stabilization, rather than regression of atherosclerosis.[50] The effects of bisphosphonates on plaque stabilization remain unknown. Intravascular ultrasound may be more useful to assess the changes in plaque volume and characteristics.[51] It is also possible that CV protective effects may depend on different bisphosphonate agents. Etidronate, a non-nitrogen-containing bisphosphonate, is considered to be the most potent inhibitor of vascular calcification,[21] but our meta-analysis did not have a sufficient number of trials that evaluated etidronate.

Our findings on MI and stroke contradict the results from epidemiologic studies.[18–20] In the Taiwan National Health Insurance database, Kang et al. found 65% lower rate of MI and 21% lower rate of stroke over 2 years among patients who received at least 1 year of continuous treatment with bisphosphonates compared with patients with acute osteoporotic fracture who did not receive bisphosphonates during the follow-up period.[18,19] In a cohort of rheumatoid arthritis patients, Wolfe et al. showed 28% lower rate of MI in ever-treated patients than never-treated patients.[20] Because patients who comply with long-term bisphosphonate treatment are more likely to have better health status and healthy behaviors than those who remain untreated during the study period, such comparison may result in more favorable findings for bisphosphonates. Due to such healthy user bias, observation studies may not be appropriate to address this question. This bias is minimal in our meta-analysis that only included RCTs.

Concerns exist about the risk of serious atrial fibrillation events associated with bisphosphonates,[23,24] attributing altered intracellular ion concentration and pro-inflammatory, pro-fibrotic, and anti-angiogenic properties as potential mechanisms.[28,52] Since these early reports,[23,24] several meta-analyses of RCTs and observational studies have been conducted to assess the risk of atrial fibrillation associated with bisphosphonates. Despite similarities in the included source studies, the conclusions were inconsistent: some reviews found an increase risk of serious or any atrial fibrillation,[26,27,30,34] whereas others did not.[25,29,31–33] In our meta-analysis that included the same phase 3 trials as previous meta-analyses,[30,34] we found that the evidence was heterogeneous and the elevated risk was mainly driven by zoledronic acid.[23] The risk was not elevated for oral bisphosphonates. Recent meta-analyses by Sharma et al.[26,27] found an elevated risk for any atrial fibrillation. However, their pooled relative risks were overestimated because they included unadjusted risks from non-randomized studies. Taken together, the body of evidence suggests that oral bisphosphonates have little effects on the risk of atrial fibrillation; the risk appears to be modestly elevated for intravenous zoledronic acid. The evidence for serious atrial fibrillation events is inconclusive: even if present, the absolute risk is small and the relative risk seems lower than previously perceived. No increased risk of stroke and CV death is also reassuring.

Our meta-analysis has several strengths. It is the most comprehensive and up-to-date summary of evidence on bisphosphonates and CV events that included the largest number of trials of various bisphosphonate agents and a range of adverse CV events. We conducted several pre-specified subgroup analyses to identify treatment effect heterogeneity and sensitivity analyses to assess the impact of study quality, influential studies, and publication bias. The results were consistent across these analyses. Since we only included RCTs, confounding or healthy user bias is unlikely.

There are also important limitations. A major challenge in conducting a systematic review that evaluates treatment-related adverse events is to find complete endpoints.[35] Because adverse CV events were rare and not anticipated at the time of conducting these trials, CV event data were available in only 58 of 170 eligible trials. Most of the excluded trials due to lack of CV data probably had very few or no events. As a result, our absolute risks of CV events might have been overestimated. On the other hand, incomplete surveillance and lack of adjudication for CV events in blinded trials might have caused non-differential misclassification of CV event status. Such outcome misclassification and intention-to-treat analysis for adverse events may have decreased statistical power to detect a small risk. In addition, most trials included generally healthy adults, which can limit the generalizability of our findings to patients with high burden of CV risk factors and comorbidities. Lastly, we had limited ability to assess the CV effects of less-commonly used bisphosphonates (e.g. etidronate and pamidronate) and in some subgroups.

Since the 2008 United States Food and Drug Administration update of safety review of bisphosphonates,[53] more studies have been published. Our meta-analysis indicates that commonly prescribed bisphosphonates (alendronate, ibandronate, risedronate, and zoledronic acid) do not provide any clinically important benefits or harms on atherosclerotic CV events, but intravenous zoledronic acid may modestly increase the risk of atrial fibrillation. Considering the large reduction of osteoporotic fractures with bisphosphonates, changes in treatment decision for osteoporosis due to CV event risk are not justified. Whether less commonly prescribed agents (e.g. etidronate) prevent atherosclerotic CV events remains to be studied in future research.

## Supporting Information

S1 PRISMA Checklist (DOC)

S1 File Text 1. Systematic search strategy. **Table 1.** Characteristics of eligible randomized controlled trials by availability of cardiovascular event data. **Table 2.** Randomized controlled trials of bisphosphonates with available data on cardiovascular events. **Table 3.** Quality of included randomized controlled trials of bisphosphonates. **Table 4.** Number of adverse cardiovascular events by bisphosphonate dose. **Table 5.** Stratified analysis of bisphosphonates and adverse cardiovascular events by study quality. **Fig 1. Assessment of study quality.** Abbreviation: CV, cardiovascular. * Study quality was evaluated in the following 7 quality standards: 1) generation of random sequence, 2) concealment of allocation, 3) blinding of patients and personnel, 4) blinding of cardiovascular outcome assessors, 5) follow-up loss (>20%) in the safety analysis, 6) completeness of cardiovascular outcome reporting, and 7) ascertainment of cardiovascular outcomes. **Fig 2. Subgroup meta-analysis of total cardiovascular events associated with use of bisphosphonates.** Abbreviations: CI, confidence interval; CV, cardiovascular; IV, intravenous; M-H, Mantel Haenszel; OR, odds ratio; PO, per os. * Heterogeneity by subgroup (P value for interaction) was assessed using meta-regression. **Fig 3. Subgroup meta-analysis of atrial fibrillation associated with use of bisphosphonates.** Abbreviations: CI, confidence interval; IV, intravenous; M-H, Mantel Haenszel; OR, odds ratio; PO, per os. * Heterogeneity by subgroup (P value for interaction) was assessed using meta-regression. **Fig 4. Subgroup meta-analysis of myocardial infarction associated with use of bisphosphonates.** Abbreviations: CI, confidence interval; IV, intravenous; M-H, Mantel Haenszel; OR, odds ratio; PO, per os. * Heterogeneity by subgroup (P value for interaction) was assessed using meta-regression. **Fig 5. Subgroup meta-analysis of stroke associated with use of bisphosphonates.** Abbreviations: CI, confidence interval; IV, intravenous; M-H, Mantel Haenszel; OR, odds ratio; PO, per os. * Heterogeneity by subgroup (P value for interaction) was assessed using meta-regression. **Fig 6. Subgroup meta-analysis of cardiovascular death associated with use of bisphosphonates.** Abbreviations: CI, confidence interval; CV, cardiovascular; IV, intravenous; M-H, Mantel Haenszel; OR, odds ratio; PO, per os. * Heterogeneity by subgroup (P value for interaction) was assessed using meta-regression.
(DOCX)
