# Supplementary material for: Bisphosphonates and Risk of Cardiovascular Events: A Meta-Analysis
Source: PLoS One. 2015 Apr 17;10(4):e0122646. doi: 10.1371/journal.pone.0122646 (PMC4401508; doi:10.1371/journal.pone.0122646)
Supplement: S1 File — Systematic search strategy. Table 1. Characteristics of eligible randomized controlled trials by availability of cardiovascular event data. Table 2. Randomized controlled trials of bisphosphonates with available data on cardiovascular events. Table 3. Quality of included randomized controlled trials of bisphosphonates. Table 4. Number of adverse cardiovascular events by bisphosphonate dose. Table 5. Stratified analysis of bisphosphonates and adverse cardiovascular events by study quality. Fig 1. Assessment of study quality. Abbreviation: CV, cardiovascular. * Study quality was evaluated in the following 7 quality standards: 1) generation of random sequence, 2) concealment of allocation, 3) blinding of patients and personnel, 4) blinding of cardiovascular outcome assessors, 5) follow-up loss (>20%) in the safety analysis, 6) completeness of cardiovascular outcome reporting, and 7) ascertainment of cardiovascular outcomes. Fig 2. Subgroup meta-analysis of total cardiovascular events associated with use of bisphosphonates. Abbreviations: CI, confidence interval; CV, cardiovascular; IV, intravenous; M-H, Mantel Haenszel; OR, odds ratio; PO, per os. * Heterogeneity by subgroup (P value for interaction) was assessed using meta-regression. Fig 3. Subgroup meta-analysis of atrial fibrillation associated with use of bisphosphonates. Abbreviations: CI, confidence interval; IV, intravenous; M-H, Mantel Haenszel; OR, odds ratio; PO, per os. * Heterogeneity by subgroup (P value for interaction) was assessed using meta-regression. Fig 4. Subgroup meta-analysis of myocardial infarction associated with use of bisphosphonates. Abbreviations: CI, confidence interval; IV, intravenous; M-H, Mantel Haenszel; OR, odds ratio; PO, per os. * Heterogeneity by subgroup (P value for interaction) was assessed using meta-regression. Fig 5. Subgroup meta-analysis of stroke associated with use of bisphosphonates. Abbreviations: CI, confidence interval; IV, intravenous; M-H, Mantel Haenszel; OR, od [file pone.0122646.s002.docx]

**Supplementary Materials to**

**Bisphosphonates and Risk of Cardiovascular Events: A Meta-Analysis**

Dae Hyun Kim, James R. Rogers, Lisa A. Fulchino, Caroline A. Kim,

Daniel H. Solomon, Seoyoung C. Kim

| **Text 1.** | Systematic Search Strategy |
| --- | --- |
| **Table 1.** | Characteristics of Eligible Randomized Controlled Trials by Availability of Cardiovascular Event Data |
| **Table 2.** | Randomized Controlled Trials of Bisphosphonates with Available Data on Cardiovascular Events |
| **Table 3.** | Quality of Included Randomized Controlled Trials of Bisphosphonates |
| **Table 4.** | Number of Adverse Cardiovascular Events by Bisphosphonate Dose |
| **Table 5.** | Stratified Analysis of Bisphosphonates and Adverse Cardiovascular Events by Study Quality |
| **Figure 1.** | Assessment of Study Quality |
| **Figure 2.** | Subgroup Meta-Analysis of Total Cardiovascular Events Associated with Use of Bisphosphonates |
| **Figure 3.** | Subgroup Meta-Analysis of Atrial Fibrillation Associated with Use of Bisphosphonates |
| **Figure 4.** | Subgroup Meta-Analysis of Myocardial Infarction Associated with Use of Bisphosphonates |
| **Figure 5.** | Subgroup Meta-Analysis of Stroke Associated with Use of Bisphosphonates |
| **Figure 6.** | Subgroup Meta-Analysis of Cardiovascular Death Associated with Use of Bisphosphonates |
| **References for Supplementary Materials** | |

**Text S1. Systematic Search Strategy**

We used the following search terms to identify randomized controlled trials of bisphosphonates. The search was initially performed on August 4, 2013, and updated on July 28, 2014.

1) MEDLINE:

Search #1: (diphosphonates[mh] OR etidron*[tiab] OR clodron*[tiab] OR tiludron*[tiab] OR pamidron*[tiab] OR neridron*[tiab] OR olpadron*[tiab] OR alendron*[tiab] OR ibandron*[tiab] OR risedron*[tiab] OR zoledron*[tiab])

Search #2: ((randomized controlled trial [pt] OR controlled clinical trial [pt] OR randomized [tiab] OR placebo [tiab] OR clinical trials as topic [mesh: noexp] OR randomly [tiab] OR trial [ti]) NOT (animals [mh] NOT humans [mh])) AND adult [mh]

Search #3: (Comment[ptyp] OR Editorial[ptyp] OR Guideline[ptyp] OR Letter[ptyp] OR Meta-Analysis[ptyp] OR Review[ptyp] OR systematic[sb])

Search #4: (#1 AND #2) NOT #3

2) EMBASE:

Search #1: (‘bisphosphonic acid derivative’:cl OR etidrona*:ab,ti OR clodron*:ab,ti OR tiludron*:ab,ti OR pamidron*:ab,ti OR neridron*:ab,ti OR olpadron*:ab,ti OR alendron*:ab,ti OR ibandron*:ab,ti OR risedron*:ab,ti OR zoledron*:ab,ti)

Search #2: ((random$:ab,ti OR factorial$:ab,ti OR crossover$:ab,ti OR 'cross over':ab,ti OR 'cross-over':ab,ti OR placebo$:ab,ti OR 'double blind':ab,ti OR 'single blind':ab,ti OR assign$:ab,ti OR allocat$:ab,ti OR volunteer$:ab,ti OR 'crossover procedure':cl OR 'double blind procedure':cl OR 'randomized controlled trial':cl OR 'single blind procedure':cl) NOT ([animals]/lim NOT [humans]/lim)) AND ([adult]/lim OR [aged]/lim)

Search #3: ([editorial]/lim OR [letter]/lim OR [meta analysis]/lim OR [systematic review]/lim OR [review]/lim)

Search #4: ((#1 AND #2) NOT #3) AND [embase]/lim

**Table S1. Characteristics of Eligible Randomized Controlled Trials by Availability of Cardiovascular Event Data**

| **Characteristics** | **RCTs with CV Event Data** | **RCTs without CV Event Data** | ***P**** |
| --- | --- | --- | --- |
| Number of eligible RCTs | 58 | 112 |  |
| Type of bisphosphonate, *n (%)* |  |  | 0.01 |
| Alendronate | 29 (50.0) | 33 (29.5) |  |
| Ibandronate | 7 (12.1) | 10 (8.9) |  |
| Risedronate | 8 (13.8) | 25 (22.3) |  |
| Zoledronic acid | 9 (15.5) | 13 (11.6) |  |
| Others^†^ | 5 (8.6) | 31 (27.7) |  |
| Sample size |  |  |  |
| Bisphosphonate group, *median (IQR)* | 158 (86, 563) | 37 (21, 87) | <0.001 |
| No bisphosphonate group, *median (IQR)* | 92 (48, 286) | 35 (20, 79) | <0.001 |
| Participant characteristics^‡^ |  |  |  |
| Mean age, *years, mean (SD)* | 63.0 (7.6) | 58.8 (11.6) | 0.005 |
| Proportion of female, *%, mean (SD)* | 84.4 (29.2) | 75.1 (30.8) | 0.06 |
| Mean weight, *kg, mean (SD)* | 64.8 (5.5) | 66.2 (8.3) | 0.48 |
| Mean body mass index, *kg/m^2^, mean (SD)* | 25.4 (1.4) | 25.0 (3.2) | 0.51 |
| Follow-up duration, *months, median (IQR)* | 24 (12, 36) | 12 (12, 24) | 0.003 |

Abbreviations: CV, cardiovascular; IQR, interquartile range; RCT, randomized controlled trial; SD, standard deviation.

* P-values were computed from two-sample t tests or Wilcoxon Ranksum tests for continuous variables and chi-square test for categorical variables.

^†^ Other bisphosphonates include clodronate, etidronate, minodronate, pamidronate, and tiludronate.

^‡^ The number of trials that reported relevant characteristics was 163 for the mean age, 164 for proportion of female, 62 for the mean weight, and 88 for the mean body mass index.

**Table S2. Randomized Controlled Trials of Bisphosphonates with Available Data on Cardiovascular Events**

| **Trial Name or Author (Year)** | **Bisphosphonate** | **Route** | **Sample Size** | | **Mean**  **Age**  *y* | **Women**  % | **Wt**  *kg* | **BMI**  *kg/m^2^* | **Sm**  % | **Study Population Characteristics** | **FU***  *m* | **Reported Events** |
| --- | --- | --- | --- | --- | --- | --- | --- | --- | --- | --- | --- | --- |
|  |  |  | *BIS* | *CTR* |  |  |  |  |  |  |  |  |
| Adami (1993)[1]^†^ | Alendronate 10-20mg/d | PO | 140 | 71 | 59 | 100 | 60 | NR | 18 | Post-menopause  Osteoporosis | 24 | AF |
| Chesnut (1995)[2]^†^ | Alendronate 5-20mg/d | PO | 94 | 31 | 63 | 100 | 63 | NR | NR | Post-menopause  Osteoporosis | 24 | AF |
| Devogelaer (1996)[3]^†^ | Alendronate 5-20mg/d | PO | 311 | 205 | 63 | 100 | 61 | 25 | 10 | Post-menopause  Osteoporosis | 36 | AF |
| Tucci (1996)[4]^†^ | Alendronate 5-20mg/d | PO | 286 | 192 | 65 | 100 | NR | 24 | NR | Post-menopause  Osteoporosis | 36 | AF |
| FIT I (1996)[5]^†^ | Alendronate 5-10mg/d | PO | 1022 | 1005 | 71 | 100 | NR | NR | 11 | Post-menopause  Osteoporosis | 36 | AF |
| Bone (1997)[6]^†^ | Alendronate 5mg/d | PO | 93 | 91 | 71 | 100 | 61 | NR | NR | Post-menopause  Osteoporosis | 24 | AF |
| EPIC (1998)[7,8]^†^ | Alendronate 2.5-5mg/d | PO | 997 | 502 | 55 | 100 | NR | 25 | 20 | Post-menopause  No osteoporosis | 24,  48 | AF, Total CV events |
| FIT II (1998)[9]^†^ | Alendronate 5-10mg/d | PO | 2214 | 2218 | 68 | 100 | NR | NR | 10 | Post-menopause  Osteoporosis | 48 | AF |
| Greenspan (1998)[10]^†^ | Alendronate 5-10mg/d | PO | 60 | 60 | 70 | 100 | 65 | 26 | NR | Post-menopause | 36 | AF |
| McClung (1998)[11]^†^ | Alendronate 5-20mg/d | PO | 265 | 90 | 52 | 100 | 64 | 24 | 37 | Post-menopause  No osteoporosis | 36 | AF |
| Saag (1998)[12]^†^ | Alendronate 5-10mg/d | PO | 318 | 159 | 55 | 70 | NR | NR | NR | Long-term steroid use | 12 | AF |
| Lindsay (1999)[13]^†^ | Alendronate 10mg/d | PO | 214 | 214 | 62 | 100 | NR | 24 | NR | Post-menopause  Osteoporosis, on HRT | 12 | AF |
| FOSIT (1999)[14]^†^ | Alendronate 10mg/d | PO | 950 | 958 | 63 | 100 | 64 | NR | NR | Post-menopause  Osteopenia/osteoporosis | 12 | AF |
| Downs (2000)[15]^†^ | Alendronate 10mg/d | PO | 118 | 58 | 65 | 100 | NR | 26 | NR | Post-menopause  Osteoporosis | 12 | AF |
| Orwoll (2000)[16]^†^ | Alendronate 10mg/d | PO | 146 | 95 | 63 | 0 | NR | 25 | 26 | Osteoporosis | 24 | AF, Total CV events |
| Bell (2002)[17]^†^ | Alendronate 10mg/d | PO | 33 | 32 | 66 | 100 | 70 | NR | NR | Post-menopause  Osteoporosis | 24 | AF |
| Greenspan (2002)[18]^†^ | Alendronate 10mg/d | PO | 164 | 163 | 79 | 100 | NR | NR | NR | Post-menopause  Osteoporosis | 24 | AF |
| Palomba (2002)[19] | Alendronate 5-10mg/d | PO | 86 | 43 | 61 | 100 | NR | 25 | NR | Surgical menopause  Osteoporosis, on ERT | 24 | MI, CV death |
| van der Poest Clement (2002)[20]^†^ | Alendronate 10mg/d | PO | 21 | 20 | 46 | 44 | NR | 25 | NR | Fracture | 12 | AF |
| Ascott-Evans (2003)[21]^†^ | Alendronate 10mg/d | PO | 95 | 49 | 57 | 100 | NR | 25 | NR | Post-menopause  Osteopenia/osteoporosis | 12 | AF |
| Greenspan (2003)[22] | Alendronate 10mg/d | PO | 93 | 93 | 72 | 100 | 70 | 28 | 6 | Post-menopause  Osteoporosis | 36 | MI |
| Hosking (2003)[23]^†^ | Alendronate 70mg/w | PO | 219 | 108 | 69 | 100 | NR | 25 | NR | Post-menopause  Osteoporosis | 12 | AF |
| Milller (2004)[24]^†^ | Alendronate 70mg/w | PO | 97 | 46 | 66 | 0 | 79 | 26 | 11 | Osteoporosis | 12 | AF |
| Chevrel (2006)[25] | Alendronate 10mg/d | PO | 31 | 33 | 37 | 39 | NR | NR | NR | Osteogenesis Imperfecta | 36 | Total CV events |
| Lems (2006)[26] | Alendronate 5-10mg/d | PO | 94 | 69 | 62 | 56 | NR | NR | NR | Rheumatoid arthritis  Low-dose prednisone | 12 | Total CV events |
| McClung (2006)[27]  Lewiecki (2007)[28] | Alendronate 70mg/w | PO | 47 | 46 | 63 | 100 | NR | 26 | NR | Post-menopause  Osteopenia/osteoporosis | 12,  24 | Total CV events |
| Bonnick (2007)[29]^†^ | Alendronate 10mg/d | PO | 563 | 138 | 66 | 100 | NR | NR | 0 | Post-menopause  Osteoporosis | 24 | AF |
| Stoch (2009)[30]^†^ | Alendronate 70mg/w | PO | 114 | 59 | 53 | 58 | NR | NR | NR | Rheumatic disorders  Long-term prednisone | 12 | AF, CV death |
| Eastell (2011)[31] | Alendronate 70mg/w | PO | 57 | 57 | 65 | 100 | NR | 25 | NR | Post-menopause  Osteoporosis | 12 | Total CV events |
| Roux (1998)[32] | Etidronate 400mg/d for 14d cycled every 3m | PO | 59 | 58 | 59 | 64 | NR | NR | NR | High-dose steroid | 12 | MI, CV death |
| Wimalawansa (1998)[33] | Etidronate 400mg/d for 14d cycled every 3m | PO | 17 | 18 | 65 | 100 | NR | 25 | NR | Post-menopause  Osteoporosis | 48 | MI, CV death |
| Ravn (1996)[34] | Ibandronate 0.25-5mg/d | PO | 150 | 30 | 64 | 100 | 67 | NR | NR | Post-menopause  Osteopenia/osteoporosis | 12 | MI, ST, CV death |
| Adami (2004)[35] | Ibandronate 1-2mg once | IV | 392 | 128 | 66 | 100 | 64 | NR | NR | Post-menopause  Osteoporosis | 12 | Total CV events |
| BONE (2004)[36]^‡^ | Ibandronate 2.5mg/d or 20mg/2d for 12 doses/3m | PO | 1954 | 975 | 69 | 100 | 67 | NR | NR | Post-menopause  Osteoporosis | 36 | AF |
| IVF (2004)[37]^‡^ | Ibandronate 0.5-1mg/3m | IV | 1911 | 949 | 67 | 100 | NR | NR | NR | Post-menopause  Osteoporosis | 36 | AF, Total CV events |
| MOBILE (2006)[38]^‡^ | Ibandronate 2.5mg/d or 100-150mg/m | PO | 1583 | - | 66 | 100 | 64 | 26 | NR | Post-menopause  Osteoporosis | 24 | AF |
| DIVA (2008)[39]^‡^ | Ibandronate PO 2.5mg/d or IV 2mg/2m or IV 3mg/3m | PO  IV | 1382 | - | 66 | 100 | 64 | 26 | NR | Post-menopause  Osteoporosis | 24 | AF |
| Hakala (2012)[40] | Ibandronate 150mg/m | PO | 68 | 72 | 64 | 100 | NR | 30 | NR | Post-menopause  No osteoporosis | 12 | ST |
| Matsumoto (2009)[41] | Minodronate 1mg/d | PO | 343 | 331 | 72 | 100 | NR | 23 | NR | Post-menopause  Osteoporosis | 24 | AF, CHD, HF |
| Eggelmeijer (1996)[42] | Pamidronate 300mg/d | PO | 54 | 51 | 50 | 68 | NR | NR | NR | Rheumatoid arthritis  Not treated with steroid | 36 | MI, CV death |
| Shetty (2006)[43] | Pamidronate 90mg once | IV | 18 | 19 | 58 | 41 | NR | 28 | NR | Total hip replacement for osteoarthritis | 60 | CHD |
| Mortensen (1998)[44]^§^ | Risedronate 5mg/d or cyclically | PO | 75 | 36 | 51 | 100 | NR | NR | NR | Post-menopause  No osteoporosis | 24 | AF, ST, CV death |
| Cohen (1999)[45]^§^ | Risedronate 2.5-5mg/d | PO | 151 | 77 | 60 | 66 | NR | NR | NR | High-dose steroid | 12 | AF, ST, CV death |
| VERT-NA (1999)[46]^§^ | Risedronate 2.5-5mg/d | PO | 1638 | 820 | 69 | 100 | 67 | NR | NR | Post-menopause  Osteoporosis | 36 | AF, ST, CV death |
| Reid (2000)[47]^§^ | Risedronate 2.5-5mg/d | PO | 194 | 96 | 59 | 62 | NR | NR | NR | High-dose steroid | 12 | AF, ST, CV death |
| VERT-MN (2000)[48]^§^ | Risedronate 2.5-5mg/d | PO | 815 | 407 | 71 | 100 | NR | NR | NR | Post-menopause  Osteoporosis | 36 | AF, ST, CV death, total CV events |
| HIP (2001)[49]^§^ | Risedronate 2.5-5mg/d | PO | 6197 | 3134 | 78 | 100 | 61 | NR | NR | Post-menopause  Osteoporosis | 36 | AF, ST, CV death |
| Shiraki (2003)[50] | Risedronate 1-5mg/d | PO | 125 | 43 | 61 | 99 | 50 | NR | NR | Post-menopause  Osteoporosis | 9 | Total CV events |
| Boonen (2009)[51] | Risedronate 35mg/w | PO | 191 | 93 | 61 | 0 | NR | 25 | NR | Osteoporosis | 24 | AF, MI |
| HORIZON-  PFT (2007)[52,53] | Zoledronic acid 5mg/y | IV | 3862 | 3852 | 73 | 100 | NR | 25 | NR | Post-menopause  Osteoporosis | 36 | AF, MI, ST, CV death |
| HORIZON-  RFT (2007)[53-55] | Zoledronic acid 5mg/y | IV | 1054 | 1057 | 75 | 76 | NR | 25 | NR | Recent surgical repair of low-trauma hip fracture | 23 | AF, MI, ST, CV death |
| Grey (2009)[56-58] | Zoledronic acid 5mg once | IV | 25 | 25 | 64 | 100 | 68 | NR | 6 | Post-menopause  Osteopenia | 24,  36,  60 | AF |
| McClung (2009)[59] | Zoledronic acid 5mg once or 5mg/y | IV | 379 | 202 | 60 | 100 | NR | 27 | NR | Post-menopause  Osteopenia | 24 | AF |
| Boonen (2012)[60] | Zoledronic acid 5mg/y | IV | 588 | 611 | 66 | 0 | NR | NR | NR | Osteoporosis | 24 | AF, CHD, MI, total CV events |
| Grey (2012)[61] | Zoledronic acid 1-5mg once | IV | 135 | 45 | 65 | 100 | 66 | NR | 1 | Post-menopause  Osteopenia | 12 | AF |
| Bai (2013)[62] | Zoledronic acid 5mg/y | IV | 242 | 241 | 57 | 100 | NR | 24 | NR | Post-menopause  Osteoporosis | 24 | Total CV events |
| Chao (2013)[63] | Zoledronic acid 5mg/y | IV | 327 | 333 | 55 | 100 | NR | 24 | NR | Post-menopause  Osteoporosis | 36 | Total CV events |
| Dalbeth (2014)[64] | Zoledronic acid 5mg/y | IV | 50 | 50 | 56 | 53 | NR | NR | NR | Tophaceous gout | 24 | Total CV events |

Abbreviations: AF, atrial fibrillation; BIS, bisphosphonate; BMI, body mass index; CHD, coronary heart disease; CTR, control; CV, cardiovascular; d, day; DIVA, Dosing IntraVenous Administration; EPIC, Early Postmenopausal Intervention Cohort study; ERT, estrogen replacement therapy; FIT, Fracture Intervention Trial; FOSIT, Fosamax International Trial; FU, follow-up; HF, heart failure; HIP, Hip Intervention Program Study; HORIZON-PFT, the Health Outcomes and Reduced Incidence with Zoledronic Acid Once Yearly Pivotal Fracture Trial; HORIZON-RFT, the Health Outcomes and Reduced Incidence with Zoledronic Acid Once Yearly Recurrent Fracture Trial; HRT, hormone replacement therapy; IV, intravenous; IVF, IntraVenous Fracture study; m, month; MI, myocardial infarction; MOBILE, Monthly Oral iBandronate in LadiEs; NR, not reported; PO, per os; Sm, smoking; ST, stroke; VERT-MN, Vertebral Efficacy with Risedronate Therapy Multinational Study; VERT-NA, Vertebral Efficacy with Risedronate Therapy North America Study; w, week; Wt, weight; y, year.

* Some trials reported data on cardiovascular events at different follow-up time.

^†^ Data on atrial fibrillation were available in the meta-analysis by Barrett-Connor et al.[65]

^‡^ Data on atrial fibrillation were available in the pooled analysis of 4 trials by Lewiecki et al.[66]

^§^ Data on atrial fibrillation, stroke, and cardiovascular death were available in the pooled analysis of 6 trials by Karam et al.[67] The sample size of bisphosphonate group and placebo group in the pooled analysis (N_BIS_=10018 and N_PLC_=5048, respectively) was larger than the sum of sample size that was originally reported in individual trials (N_BIS_=9070 and N_PLC_=4570, respectively).

**Table S3. Quality of Included Randomized Controlled Trials of Bisphosphonates***

| **Trial Name or Author (Year)** | **Generation of Random Sequence** | **Concealment of Allocation** | **Blinding of Participants and Personnel** | **Blinding of**  **CV Outcome Assessors** | **Adequacy of Follow-Up in**  **Safety Analysis** | **CV Event Reporting** | **Ascertainment of CV Events** |
| --- | --- | --- | --- | --- | --- | --- | --- |
| Adami (1993)[1]^†^ | Inadequate | Inadequate | Adequate | Adequate | Adequate | Incomplete | Inadequate |
| Chesnut (1995)[2]^†^ | Inadequate | Inadequate | Adequate | Adequate | Adequate | Incomplete | Inadequate |
| Devogelaer (1996)[3]^†^ | Inadequate | Inadequate | Adequate | Adequate | Adequate | Incomplete | Inadequate |
| Tucci (1996)[4]^†^ | Inadequate | Inadequate | Adequate | Adequate | Adequate | Incomplete | Inadequate |
| FIT I (1996)[5]^†^ | Inadequate | Adequate | Adequate | Adequate | Adequate | Incomplete | Adequate |
| Bone (1997)[6]^†^ | Inadequate | Inadequate | Adequate | Adequate | Inadequate | Incomplete | Inadequate |
| EPIC (1998)[7,8]^†^ | Inadequate | Inadequate | Adequate | Adequate | Adequate | Incomplete | Inadequate |
| FIT II (1998)[9]^†^ | Inadequate | Adequate | Adequate | Adequate | Adequate | Incomplete | Adequate |
| Greenspan (1998)[10]^†^ | Inadequate | Inadequate | Adequate | Adequate | Inadequate | Incomplete | Inadequate |
| McClung (1998)[11]^†^ | Inadequate | Inadequate | Adequate | Adequate | Inadequate | Incomplete | Inadequate |
| Saag (1998)[12]^†^ | Inadequate | Inadequate | Adequate | Adequate | Adequate | Incomplete | Inadequate |
| Lindsay (1999)[13]^†^ | Inadequate | Inadequate | Adequate | Adequate | Adequate | Incomplete | Inadequate |
| FOSIT (1999)[14]^†^ | Inadequate | Inadequate | Adequate | Adequate | Adequate | Incomplete | Inadequate |
| Downs (2000)[15]^†^ | Inadequate | Inadequate | Adequate | Adequate | Inadequate | Incomplete | Inadequate |
| Orwoll (2000)[16]^†^ | Inadequate | Inadequate | Adequate | Adequate | Adequate | Incomplete | Inadequate |
| Bell (2002)[17]^†^ | Inadequate | Inadequate | Adequate | Adequate | Adequate | Incomplete | Inadequate |
| Greenspan (2002)[18]^†^ | Adequate | Inadequate | Adequate | Adequate | Inadequate | Incomplete | Inadequate |
| Palomba (2002)[19] | Adequate | Inadequate | Adequate | Adequate | Adequate | Incomplete | Inadequate |
| van der Poest (2002)[20]^†^ | Inadequate | Inadequate | Adequate | Adequate | Adequate | Incomplete | Inadequate |
| Ascott-Evans (2003)[21]^†^ | Adequate | Inadequate | Adequate | Adequate | Adequate | Incomplete | Inadequate |
| Greenspan (2003)[22] | Adequate | Adequate | Adequate | Adequate | Adequate | Incomplete | Inadequate |
| Hosking (2003)[23]^†^ | Adequate | Inadequate | Adequate | Adequate | Inadequate | Incomplete | Inadequate |
| Milller (2004)[24]^†^ | Adequate | Inadequate | Adequate | Adequate | Adequate | Incomplete | Inadequate |
| Chevrel (2006)[25] | Adequate | Inadequate | Adequate | Adequate | Adequate | Incomplete | Inadequate |
| Lems (2006)[26] | Inadequate | Inadequate | Adequate | Adequate | Adequate | Incomplete | Inadequate |
| McClung (2006)[27]  Lewiecki (2007)[28] | Inadequate | Inadequate | Inadequate | Inadequate | Adequate | Incomplete | Inadequate |
| Bonnick (2007)[29]^†^ | Inadequate | Inadequate | Adequate | Adequate | Adequate | Incomplete | Inadequate |
| Stoch (2009)[30]^†^ | Inadequate | Inadequate | Adequate | Adequate | Adequate | Incomplete | Inadequate |
| Eastell (2011)[31] | Inadequate | Inadequate | Adequate | Adequate | Adequate | Incomplete | Inadequate |
| Roux (1998)[32] | Inadequate | Inadequate | Adequate | Adequate | Adequate | Incomplete | Inadequate |
| Wimalawansa (1998)[33] | Adequate | Inadequate | Inadequate | Inadequate | Inadequate | Complete | Inadequate |
| Ravn (1996)[34] | Inadequate | Inadequate | Adequate | Adequate | Adequate | Incomplete | Inadequate |
| Adami (2004)[35] | Inadequate | Inadequate | Adequate | Adequate | Adequate | Incomplete | Inadequate |
| BONE (2004)[36]^‡^ | Inadequate | Inadequate | Adequate | Adequate | Adequate | Incomplete | Inadequate |
| IVF (2004)[37]^‡^ | Inadequate | Inadequate | Adequate | Adequate | Adequate | Incomplete | Inadequate |
| MOBILE (2006)[38]^‡^ | Inadequate | Inadequate | Adequate | Adequate | Adequate | Incomplete | Inadequate |
| DIVA (2008)[39]^‡^ | Inadequate | Adequate | Adequate | Adequate | Adequate | Incomplete | Inadequate |
| Hakala (2012)[40] | Inadequate | Inadequate | Adequate | Adequate | Adequate | Incomplete | Inadequate |
| Matsumoto (2009)[41] | Adequate | Inadequate | Adequate | Adequate | Adequate | Incomplete | Inadequate |
| Eggelmeijer (1996)[42] | Inadequate | Inadequate | Adequate | Adequate | Inadequate | Incomplete | Inadequate |
| Shetty (2006)[43] | Adequate | Inadequate | Adequate | Adequate | Inadequate | Incomplete | Inadequate |
| Mortensen (1998)[44]^§^ | Inadequate | Inadequate | Adequate | Adequate | Inadequate | Incomplete | Inadequate |
| Cohen (1999)[45]^§^ | Inadequate | Inadequate | Adequate | Adequate | Adequate | Incomplete | Inadequate |
| VERT-NA (1999)[46]^§^ | Adequate | Adequate | Adequate | Adequate | Adequate | Incomplete | Inadequate |
| Reid (2000)[47]^§^ | Inadequate | Inadequate | Adequate | Adequate | Adequate | Incomplete | Inadequate |
| VERT-MN (2000)[48]^§^ | Inadequate | Inadequate | Adequate | Adequate | Inadequate | Incomplete | Inadequate |
| HIP (2001)[49]^§^ | Inadequate | Inadequate | Adequate | Adequate | Adequate | Incomplete | Inadequate |
| Shiraki (2003)[50] | Inadequate | Inadequate | Adequate | Adequate | Adequate | Incomplete | Inadequate |
| Boonen (2009)[51] | Inadequate | Inadequate | Adequate | Adequate | Adequate | Incomplete | Inadequate |
| HORIZON-  PFT (2007)[52,53] | Inadequate | Inadequate | Adequate | Adequate | Adequate | Complete | Adequate |
| HORIZON-  RFT (2007)[53-55] | Inadequate | Adequate | Adequate | Adequate | Adequate | Complete | Inadequate |
| Grey (2009)[56-58] | Adequate | Adequate | Adequate | Adequate | Inadequate | Incomplete | Inadequate |
| McClung (2009)[59] | Adequate | Adequate | Adequate | Adequate | Adequate | Incomplete | Inadequate |
| Boonen (2012)[60] | Adequate | Inadequate | Adequate | Adequate | Adequate | Incomplete | Inadequate |
| Grey (2012)[61] | Adequate | Inadequate | Adequate | Adequate | Adequate | Incomplete | Inadequate |
| Bai (2013)[62] | Inadequate | Inadequate | Inadequate | Inadequate | Inadequate | Incomplete | Inadequate |
| Chao (2013)[63] | Inadequate | Inadequate | Inadequate | Inadequate | Inadequate | Incomplete | Inadequate |
| Dalbeth (2014)[64] | Adequate | Adequate | Adequate | Adequate | Inadequate | Incomplete | Inadequate |

Abbreviations: CV, cardiovascular; DIVA, Dosing IntraVenous Administration; EPIC, Early Postmenopausal Intervention Cohort study; FIT, Fracture Intervention Trial; FOSIT, Fosamax International Trial; HIP, Hip Intervention Program Study; HORIZON-PFT, the Health Outcomes and Reduced Incidence with Zoledronic Acid Once Yearly Pivotal Fracture Trial; HORIZON-RFT, the Health Outcomes and Reduced Incidence with Zoledronic Acid Once Yearly Recurrent Fracture Trial; IVF, IntraVenous Fracture study; MOBILE, Monthly Oral iBandronate in LadiEs; VERT-MN, Vertebral Efficacy with Risedronate Therapy Multinational Study; VERT-NA, Vertebral Efficacy with Risedronate Therapy North America Study.

* Study quality was evaluated in the following 7 quality standards: 1) generation of random sequence, 2) concealment of allocation, 3) blinding of patients and personnel, 4) blinding of cardiovascular outcome assessors, 5) follow-up loss (>20%) in the safety analysis, 6) completeness of cardiovascular outcome reporting, and 7) ascertainment of cardiovascular outcomes.

**Table S4. Number of Adverse Cardiovascular Events by Bisphosphonate Dose***

|  | **Total CV Events** | | **Atrial Fibrillation** | | **MI** | | **Stroke** | | **CV Death** | |
| --- | --- | --- | --- | --- | --- | --- | --- | --- | --- | --- |
| **Dose**^†^ | **N** | **N_events_ / N_total_ (%)** | **N** | **N_events_ / N_total_ (%)** | **N** | **N_events_ / N_total_ (%)** | **N** | **N_events_ / N_total_ (%)** | **N** | **N_events_ / N_total_ (%)** |
| **Alendronate** | | | | | | | | | | |
| Placebo | 5 | 111 / 733 (15.1) | 15 | 13 / 2173 (0.6) | 2 | 1 / 136 (0.7) | 0 | - | 2 | 0 / 102 (0) |
| PO 2.5mg/d | 1 | 64 / 330 (19.4) | 0 | - | 0 | - | 0 | - | 0 | - |
| PO 5mg/d | 1 | 59 / 333 (17.7) | 1 | 0 / 93 (0) | 1 | 1/ 44 (2.3) | 0 | - | 1 | 1 / 44 (2.3) |
| PO 10mg/d | 4 | 33 / 281 (11.7) | 13 | 15 / 2802 (0.5) | 1 | 2 / 135 (1.5) | 0 | - | 2 | 2 / 156 (1.3) |
| PO 20mg/d | 0 | - | 1 | 0 / 72 (0) | 0 | - | 0 | - | 0 | - |
| **Ibandronate** | | | | | | | | | | |
| Placebo | 2 | 73 / 1077 (6.8) | 2 | 18 / 1924 (0.9) | 1 | 0 / 30 (0) | 2 | 1 / 102 (1.0) | 1 | 1 / 30 (3.3) |
| PO 0.25-1mg/d or  IV 0.5-1mg/3m | 2 | 152 / 2303 (6.6) | 1 | 18 / 1911 (0.9) | 1 | 0 / 90 (0) | 1 | 0 / 90 (0) | 1 | 0 / 90 (0) |
| PO 2.5-3mg/d | 0 | - | 3 | 29 / 3606 (0.8) | 1 | 1 / 30 (3.3) | 1 | 0 / 30 (0) | 1 | 0 / 30 (0) |
| PO 5mg/d or  IV 1mg/m | 0 | - | 2 | 10 / 1313 (0.8) | 1 | 0 / 30 (0) | 2 | 1 / 98 (1.0) | 1 | 0 / 30 (0) |
| **Risedronate** | | | | | | | | | | |
| Placebo | 2 | 40 / 450 (8.8) | 7 | 97 / 10189 (1.0) | 1 | 3 / 93 (3.2) | 6 | 77 / 5048 (1.5) | 6 | 96 / 5048 (1.9) |
| PO 1mg/d | 1 | 0 / 39 (0) | 0 | - | 0 | - | 0 | - | 0 | - |
| PO 2.5mg/d | 2 | 30 / 447 (6.7) | 5 | 66 / 4998 (1.3) | 0 | - | 5 | 71 / 4998 (1.4) | 5 | 83 / 4998 (1.7) |
| PO 5mg/d | 2 | 38 / 454 (8.4) | 7 | 72 / 5211 (1.4) | 1 | 2 / 191 (1.0) | 6 | 70 / 5020 (1.4) | 6 | 80 / 5020 (1.6) |
| **Zoledronic acid** | | | | | | | | | | |
| Placebo | 4 | 48 / 1235 (3.9) | 5 | 105 / 5767 (1.8) | 3 | 64 / 5520 (1.2) | 2 | 126 / 4909 (2.6) | 2 | 89 / 4909 (1.8) |
| IV 1mg/y | 0 | - | 1 | 0 / 45 (0) | 0 | - | 0 | - | 0 | - |
| IV 2.5mg/y | 0 | - | 1 | 0 / 45 (0) | 0 | - | 0 | - | 0 | - |
| IV 5mg/y | 4 | 49 / 1207 (4.1) | 5 | 130 / 5928 (2.2) | 3 | 60 / 5504 (1.1) | 2 | 133 / 4916 (2.7) | 2 | 75 / 4916 (1.5) |

Abbreviations: CV, cardiovascular; d, day; IV, intravenous; m, month; MI, myocardial infarction; PO, per os; y, year.

* Trials that did not report outcome data for specific doses were excluded.

^†^ Average daily dose was calculated for oral bisphosphonate regimen.

**Table S5. Stratified Analysis of Bisphosphonates and Adverse Cardiovascular Events by Study Quality***

|  | **Total CV Events** | | **Atrial Fibrillation** | | **MI** | | **Stroke** | | **CV Death** | |
| --- | --- | --- | --- | --- | --- | --- | --- | --- | --- | --- |
| **Quality Standard** | **N**^†^ | **M-H OR (95% CI)** | **N**^†^ | **M-H OR (95% CI)** | **N**^†^ | **M-H OR (95% CI)** | **N**^†^ | **M-H OR (95% CI)** | **N**^†^ | **M-H OR (95% CI)** |
| **Generation of Random Sequence** | | | | | | | | | | |
| Adequate | 3 | 1.09 (0.69, 1.71) | 3 | 1.22 (0.55, 2.71) | 4 | 3.34 (1.09, 10.2) | 0 | - | 2 | 2.20 (0.22, 21.7) |
| Inadequate | 11 | 0.97 (0.82, 1.14) | 18 | 1.07 (0.92, 1.25) | 6 | 0.82 (0.58, 1.17) | 5 | 0.99 (0.82, 1.19) | 7 | 0.87 (0.72, 1.06) |
| *P for interaction* |  | 0.66 |  | 0.76 |  | 0.05 |  | - |  | 0.46 |
| **Concealment of Allocation** | | | | | | | | | | |
| Adequate | 1 | 1.00 (0.37, 2.73) | 3 | 1.13 (0.86, 1.48) | 2 | 0.84 (0.42, 1.67) | 1 | 1.21 (0.78, 1.88) | 1 | 0.69 (0.45, 1.07) |
| Inadequate | 13 | 0.98 (0.84, 1.15) | 18 | 1.05 (0.88, 1.27) | 7 | 1.01 (0.69, 1.47) | 4 | 0.95 (0.77, 1.16) | 8 | 0.94 (0.75, 1.17) |
| *P for interaction* |  | 0.97 |  | 0.70 |  | 0.70 |  | 0.40 |  | 0.30 |
| **Blinding of Participants and Personnel** | | | | | | | | | | |
| Adequate | 11 | 0.98 (0.83, 1.15) | 21 | 1.08 (0.92, 1.25) | 9 | 0.95 (0.68, 1.33) | 5 | 0.99 (0.82, 1.19) | 8 | 0.88 (0.72, 1.06) |
| Inadequate | 3 | 1.00 (0.43, 2.33) | 0 | - | 1 | 3.17 (0.12, 83.2) | 0 | - | 1 | 3.17 (0.12, 83.2) |
| *P for interaction* |  | 0.98 |  | - |  | 0.48 |  | - |  | 0.47 |
| **Blinding of CV Outcome Assessor** | | | | | | | | | | |
| Adequate | 11 | 0.98 (0.83, 1.15) | 21 | 1.08 (0.92, 1.25) | 9 | 0.95 (0.68, 1.33) | 5 | 0.99 (0.82, 1.19) | 8 | 0.88 (0.72, 1.06) |
| Inadequate | 3 | 1.00 (0.43, 2.33) | 0 | - | 1 | 3.17 (0.12, 83.2) | 0 | - | 1 | 3.17 (0.12, 83.2) |
| *P for interaction* |  | 0.98 |  | - |  | 0.48 |  | - |  | 0.47 |
| **Loss to Follow-Up in Safety Analysis** | | | | | | | | | | |
| Adequate | 10 | 0.99 (0.83, 1.18) | 15 | 1.12 (0.94, 1.35) | 8 | 0.94 (0.67, 1.31) | 4 | 1.04 (0.82, 1.33) | 6 | 0.89 (0.66, 1.21) |
| Inadequate | 4 | 0.92 (0.65, 1.32) | 6 | 0.98 (0.74, 1.29) | 2 | 3.00 (0.30, 29.7) | 1 | 0.92 (0.70, 1.22) | 3 | 0.87 (0.68, 1.12) |
| *P for interaction* |  | 0.69 |  | 0.43 |  | 0.34 |  | 0.61 |  | 0.96 |
| **CV Event Reporting** | | | | | | | | | | |
| Complete | 0 | - | 2 | 1.23 (0.94, 1.61) | 3 | 0.84 (0.58, 1.21) | 2 | 1.05 (0.82, 1.35) | 3 | 0.90 (0.66, 1.23) |
| Incomplete | 14 | 0.98 (0.84, 1.14) | 19 | 1.01 (0.84, 1.22) | 7 | 1.79 (0.79, 4.03) | 3 | 0.91 (0.69, 1.20) | 6 | 0.87 (0.68, 1.11) |
| *P for interaction* |  | - |  | 0.26 |  | 0.21 |  | 0.55 |  | 0.94 |
| **Ascertainment of CV Events** | | | | | | | | | | |
| Adequate | 0 | - | 3 | 1.22 (0.97, 1.52) | 1 | 0.82 (0.55, 1.30) | 1 | 0.99 (0.73, 1.33) | 1 | 1.18 (0.74, 1.88) |
| Inadequate | 14 | 0.98 (0.84, 1.14) | 18 | 0.97 (0.78, 1.19) | 9 | 1.17 (0.70, 1.96) | 4 | 0.99 (0.78, 1.25) | 8 | 0.83 (0.67, 1.02) |
| *P for interaction* |  | - |  | 0.16 |  | 0.58 |  | 0.98 |  | 0.21 |

Abbreviations: CI, confidence interval; CV, cardiovascular; M-H, Mantel Haenszel; MI, myocardial infarction; OR, odds ratio

* Heterogeneity by study quality standards (*P* value for interaction) was assessed using meta-regression.

^†^ Trials with no events were excluded.

**Figure S1. Assessment of Study Quality***

Abbreviation: CV, cardiovascular.

* Study quality was evaluated in the following 7 quality standards: 1) generation of random sequence, 2) concealment of allocation, 3) blinding of patients and personnel, 4) blinding of cardiovascular outcome assessors, 5) follow-up loss (>20%) in the safety analysis, 6) completeness of cardiovascular outcome reporting, and 7) ascertainment of cardiovascular outcomes.

**Figure S2. Subgroup Meta-Analysis of Total Cardiovascular Events Associated with Use of Bisphosphonates***

Abbreviations: CI, confidence interval; CV, cardiovascular; IV, intravenous; M-H, Mantel Haenszel; OR, odds ratio; PO, per os.

* Heterogeneity by subgroup (*P* value for interaction) was assessed using meta-regression.

**Figure S3. Subgroup Meta-Analysis of Atrial Fibrillation Associated with Use of Bisphosphonates***

Abbreviations: CI, confidence interval; IV, intravenous; M-H, Mantel Haenszel; OR, odds ratio; PO, per os.

* Heterogeneity by subgroup (*P* value for interaction) was assessed using meta-regression.

**Figure S4. Subgroup Meta-Analysis of Myocardial Infarction Associated with Use of Bisphosphonates***

Abbreviations: CI, confidence interval; IV, intravenous; M-H, Mantel Haenszel; OR, odds ratio; PO, per os.

* Heterogeneity by subgroup (*P* value for interaction) was assessed using meta-regression.

**Figure S5. Subgroup Meta-Analysis of Stroke Associated with Use of Bisphosphonates***

Abbreviations: CI, confidence interval; IV, intravenous; M-H, Mantel Haenszel; OR, odds ratio; PO, per os.

* Heterogeneity by subgroup (*P* value for interaction) was assessed using meta-regression.

**Figure S6. Subgroup Meta-Analysis of Cardiovascular Death Associated with Use of Bisphosphonates***

Abbreviations: CI, confidence interval; CV, cardiovascular; IV, intravenous; M-H, Mantel Haenszel; OR, odds ratio; PO, per os.

* Heterogeneity by subgroup (*P* value for interaction) was assessed using meta-regression.

**References for Supplementary Materials**

1. Adami S, Baroni MC, Broggini M, Carratelli L, Caruso I, et al. (1993) Treatment of postmenopausal osteoporosis with continuous daily oral alendronate in comparison with either placebo or intranasal salmon calcitonin. Osteoporos Int 3(Suppl 3): S21-27.

2. Chesnut Iii CH, McClung MR, Ensrud KE, Bell NH, Genant HK, et al. (1995) Alendronate treatment of the postmenopausal osteoporotic woman: Effect of multiple dosages on bone mass and bone remodeling. Am J Med 99: 144-152.

3. Devogelaer JP, Broll H, Correa-Rotter R, Cumming DC, De Deuxchaisnes CN, et al. (1996) Oral alendronate induces progressive increases in bone mass of the spine, hip, and total body over 3 years in postmenopausal women with osteoporosis. Bone 18: 141-150.

4. Tucci JR, Tonino RP, Emkey RD, Peverly CA, Kher U, et al. (1996) Effect of three years of oral alendronate treatment in postmenopausal women with osteoporosis. Am J Med 101: 488-501.

5. Black DM, Cummings SR, Karpf DB, Cauley JA, Thompson DE, et al. (1996) Randomised trial of effect of alendronate on risk of fracture in women with existing vertebral fractures. Lancet 348: 1535-1541.

6. Bone HG, Downs Jr RW, Tucci JR, Harris ST, Weinstein RS, et al. (1997) Dose-response relationships for alendronate treatment in osteoporotic elderly women. J Clin Endocrinol Metab 82: 265-274.

7. Hosking D, Chilvers CE, Christiansen C, Ravn P, Wasnich R, et al. (1998) Prevention of bone loss with alendronate in postmenopausal women under 60 years of age. Early Postmenopausal Intervention Cohort Study Group. N Engl J Med 338: 485-492.

8. Ravn P, Bidstrup M, Wasnich RD, Davis JW, McClung MR, et al. (1999) Alendronate and estrogen-progestin in the long-term prevention of bone loss: Four-year results from the early postmenopausal intervention cohort study: A randomized, controlled trial. Ann Intern Med 131: 935-942.

9. Cummings SR, Black DM, Thompson DE, Applegate WB, Barrett-Connor E, et al. (1998) Effect of alendronate on risk of fracture in women with low bone density but without vertebral fractures. Results from the fracture intervention trial. JAMA 280: 2077-2082.

10. Greenspan SL, Parker RA, Ferguson L, Rosen HN, Maitland-Ramsey L, et al. (1998) Early changes in biochemical markers of bone turnover predict the long- term response to alendronate therapy in representative elderly women: A randomized clinical trial. J Bone Miner Res 13: 1431-1438.

11. McClung M, Clemmesen B, Daifotis A, Gilchrist NL, Eisman J, et al. (1998) Alendronate prevents postmenopausal bone loss in women without osteoporosis. A double-blind, randomized, controlled trial. Alendronate Osteoporosis Prevention Study Group. Ann Intern Med 128: 253-261.

12. Saag KG, Emkey R, Schnitzer TJ, Brown JP, Hawkins F, et al. (1998) Alendronate for the prevention and treatment of glucocorticoid-induced osteoporosis. Glucocorticoid-Induced Osteoporosis Intervention Study Group. N Engl J Med 339: 292-299.

13. Lindsay R, Cosman F, Lobo RA, Walsh BW, Harris ST, et al. (1999) Addition of alendronate to ongoing hormone replacement therapy in the treatment of osteoporosis: A randomized, controlled clinical trial. J Clin Endocrinol Metab 84: 3076-3081.

14. Pols HA, Felsenberg D, Hanley DA, Stepan J, Munoz-Torres M, et al. (1999) Multinational, placebo-controlled, randomized trial of the effects of alendronate on bone density and fracture risk in postmenopausal women with low bone mass: results of the FOSIT study. Fosamax International Trial Study Group. Osteoporos Int 9: 461-468.

15. Downs Jr RW, Bell NH, Ettinger MP, Walsh BW, Favus MJ, et al. (2000) Comparison of alendronate and intranasal calcitonin for treatment of osteoporosis in postmenopausal women. J Clin Endocrinol Metab 85: 1783-1788.

16. Orwoll E, Ettinger M, Weiss S, Miller P, Kendler D, et al. (2000) Alendronate for the treatment of osteoporosis in men. N Engl J Med 343: 604-610.

17. Bell NH, Bilezikian JP, Bone Iii HG, Kaur A, Maragoto A, et al. (2002) Alendronate increases bone mass and reduces bone markers in postmenopausal African-American women. J Clin Endocrinol Metab 87: 2792-2797.

18. Greenspan SL, Schneider DL, McClung MR, Miller PD, Schnitzer TJ, et al. (2002) Alendronate improves bone mineral density in elderly women with osteoporosis residing in long-term care facilities: A randomized, double-blind, placebo-controlled trial. Ann Intern Med 136: 742-746.

19. Palomba S, Orio Jr F, Colao A, Carlo CD, Sena T, et al. (2002) Effect of estrogen replacement plus low-dose alendronate treatment on bone density in surgically postmenopausal women with osteoporosis. J Clin Endocrinol Metab 87: 1502-1508.

20. Van Der Poest Clement E, Van Engeland M, Ader H, Roos JC, Patka P, et al. (2002) Alendronate in the prevention of bone loss after a fracture of the lower leg. J Bone Miner Res 17: 2247-2255.

21. Ascott-Evans BH, Guanabens N, Kivinen S, Stuckey BGA, Magaril CH, et al. (2003) Alendronate prevents loss of bone density associated with discontinuation of hormone replacement therapy: A randomized controlled trial. Arch Intern Med 163: 789-794.

22. Greenspan SL, Resnick NM, Parker RA (2003) Combination Therapy with Hormone Replacement and Alendronate for Prevention of Bone Loss in Elderly Women: A Randomized Controlled Trial. JAMA 289: 2525-2533.

23. Hosking D, Adami S, Felsenberg D, Andia JC, Valimaki M, et al. (2003) Comparison of change in bone resorption and bone mineral density with once-weekly alendronate and daily risedronate: A randomised, placebo-controlled study. Curr Med Res Opin 19: 383-394.

24. Miller PD, Schnitzer T, Emkey R, Orwoll E, Rosen C, et al. (2004) Weekly oral alendronic acid in male osteoporosis. Clin Drug Invest 24: 333-341.

25. Chevrel G, Schott AM, Fontanges E, Charrin JE, Lina-Granade G, et al. (2006) Effects of oral alendronate on BMD in adult patients with osteogenesis imperfecta: A 3-year randomized placebo-controlled trial. J Bone Miner Res 21: 300-306.

26. Lems WF, Lodder MC, Lips P, Bijlsma JWJ, Geusens P, et al. (2006) Positive effect of alendronate on bone mineral density and markers of bone turnover in patients with rheumatoid arthritis on chronic treatment with low-dose prednisone: A randomized, double-blind, placebo-controlled trial. Osteoporos Int 17: 716-723.

27. McClung MR, Michael Lewiecki E, Cohen SB, Bolognese MA, Woodson GC, et al. (2006) Denosumab in postmenopausal women with low bone mineral density. N Engl J Med 354: 821-831.

28. Lewiecki EM, Miller PD, McClung MR, Cohen SB, Bolognese MA, et al. (2007) Two-year treatment with denosumab (AMG 162) in a randomized phase 2 study of postmenopausal women with low BMD. J Bone Miner Res 22: 1832-1841.

29. Bonnick S, Broy S, Kaiser F, Teutsch C, Rosenberg E, et al. (2007) Treatment with alendronate plus calcium, alendronate alone, or calcium alone for postmenopausal low bone mineral density. Curr Med Res Opin 23: 1341-1349.

30. Stoch SA, Saag KG, Greenwald M, Sebba AI, Cohen S, et al. (2009) Once-weekly oral alendronate 70 mg in patients with glucocorticoid-induced bone loss: A 12-month randomized, placebo-controlled clinical trial. J Rheumatol 36: 1705-1714.

31. Eastell R, Nagase S, Ohyama M, Small M, Sawyer J, et al. (2011) Safety and efficacy of the cathepsin K inhibitor ONO-5334 in postmenopausal osteoporosis: The OCEAN study. J Bone Miner Res 26: 1303-1312.

32. Roux C, Oriente P, Laan R, Hughes RA, Ittner J, et al. (1998) Randomized trial of effect of cyclical etidronate in the prevention of corticosteroid-induced bone loss. J Clin Endocrinol Metab 83: 1128-1133.

33. Wimalawansa SJ (1998) A four-year randomized controlled trial of hormone replacement and bisphosphonate, alone or in combination, in women with postmenopausal osteoporosis. Am J Med 104: 219-226.

34. Ravn P, Clemmesen B, Riis BJ, Christiansen C (1996) The effect on bone mass and bone markers of different doses of ibandronate: A new bisphosphonate for prevention and treatment of postmenopausal osteoporosis: A 1-year, randomized, double-blind, placebo-controlled dose-finding study. Bone 19: 527-533.

35. Adami S, Felsenberg D, Christiansen C, Robinson J, Lorenc RS, et al. (2004) Efficacy and safety of ibandronate given by intravenous injection once every 3 months. Bone 34: 881-889.

36. Chesnut IC, Skag A, Christiansen C, Recker R, Stakkestad JA, et al. (2004) Effects of oral ibandronate administered daily or intermittently on fracture risk in postmenopausal osteoporosis. J Bone Miner Res 19: 1241-1249.

37. Recker R, Stakkestad JA, Chesnut Iii CH, Christiansen C, Skag A, et al. (2004) Insufficiently dosed intravenous ibandronate injections are associated with suboptimal antifracture efficacy in postmenopausal osteoporosis. Bone 34: 890-899.

38. Reginster JY, Adami S, Lakatos P, Greenwald M, Stepan JJ, et al. (2006) Efficacy and tolerability of once-monthly oral ibandronate in postmenopausal osteoporosis: 2 Year results from the MOBILE study. Ann Rheum Dis 65: 654-661.

39. Eisman JA, Civitelli R, Adami S, Czerwinski E, Recknor C, et al. (2008) Efficacy and tolerability of intravenous ibandronate injections in postmenopausal osteoporosis: 2-Year results from the DIVA study. J Rheumatol 35: 488-497.

40. Hakala M, Kroger H, Valleala H, Hienonen-Kempas T, Lehtonen-Veromaa M, et al. (2012) Once-monthly oral ibandronate provides significant improvement in bone mineral density in postmenopausal women treated with glucocorticoids for inflammatory rheumatic diseases: A 12-month, randomized, double-blind, placebo-controlled trial. Scand J Rheumatol 41: 260-266.

41. Matsumoto T, Hagino H, Shiraki M, Fukunaga M, Nakano T, et al. (2009) Effect of daily oral minodronate on vertebral fractures in Japanese postmenopausal women with established osteoporosis: a randomized placebo-controlled double-blind study. Osteoporos Int 20: 1429-1437.

42. Eggelmeijer F, Papapoulos SE, Van Paassen HC, Dijkmans BAC, Valkema R, et al. (1996) Increased bone mass with pamidronate treatment in rheumatoid arthritis: Results of a three-year randomized, double-blind trial. Arthritis Rheum 39: 396-402.

43. Shetty N, Hamer AJ, Stockley I, Eastell R, Willkinson JM (2006) Clinical and radiological outcome of total hip replacement five years after pamidronate therapy. A trial extension. J Bone Joint Surg Br 88: 1309-1315.

44. Mortensen L, Charles P, Bekker PJ, Digennaro J, Johnston Jr CC (1998) Risedronate increases bone mass in an early postmenopausal population: Two years of treatment plus one year of follow-up. J Clin Endocrinol Metab 83: 396-402.

45. Cohen S, Levy RM, Keller M, Boling E, Emkey RD, et al. (1999) Risedronate therapy prevents corticosteroid-induced bone loss: A twelve- month, multicenter, randomized, double-blind, placebo-controlled, parallel- group study. Arthritis Rheum 42: 2309-2318.

46. Harris ST, Watts NB, Genant HK, McKeever CD, Hangartner T, et al. (1999) Effects of risedronate treatment on vertebral and nonvertebral fractures in women with postmenopausal osteoporosis: a randomized controlled trial. Vertebral Efficacy With Risedronate Therapy (VERT) Study Group. JAMA 282: 1344-1352.

47. Reid DM, Hughes RA, Laan RF, Sacco-Gibson NA, Wenderoth DH, et al. (2000) Efficacy and safety of daily risedronate in the treatment of corticosteroid-induced osteoporosis in men and women: a randomized trial. European Corticosteroid-Induced Osteoporosis Treatment Study. J Bone Miner Res 15: 1006-1013.

48. Reginster J, Minne HW, Sorensen OH, Hooper M, Roux C, et al. (2000) Randomized trial of the effects of risedronate on vertebral fractures in women with established postmenopausal osteoporosis. Vertebral Efficacy with Risedronate Therapy (VERT) Study Group. Osteoporos Int 11: 83-91.

49. McClung MR, Geusens P, Miller PD, Zippel H, Bensen WG, et al. (2001) Effect of risedronate on the risk of hip fracture in elderly women. Hip Intervention Program Study Group. N Engl J Med 344: 333-340.

50. Shiraki M, Fukunaga M, Kushida K, Kishimoto H, Taketani Y, et al. (2003) A double-blind dose-ranging study of risedronate in Japanese patients with osteoporosis (a study by the Risedronate Late Phase II Research Group). Osteoporos Int 14: 225-234.

51. Boonen S, Orwoll ES, Wenderoth D, Stoner KJ, Eusebio R, et al. (2009) Once-weekly risedronate in men with osteoporosis: Results of a 2-Year, placebo-controlled, double-blind, multicenter study. J Bone Miner Res 24: 719-725.

52. Black DM, Delmas PD, Eastell R, Reid IR, Boonen S, et al. (2007) Once-yearly zoledronic acid for treatment of postmenopausal osteoporosis. N Engl J Med 356: 1809-1822.

53. Boonen S, Black DM, Colon-Emeric CS, Eastell R, Magaziner JS, et al. (2010) Efficacy and safety of a once-yearly intravenous zoledronic acid 5 mg for fracture prevention in elderly postmenopausal women with osteoporosis aged 75 and older. J Am Geriatr Soc 58: 292-299.

54. Boonen S, Orwoll E, Magaziner J, Colon-Emeric CS, Adachi JD, et al. (2011) Once-yearly zoledronic acid in older men compared with women with recent hip fracture. J Am Geriatr Soc 59: 2084-2090.

55. Lyles KW, Colon-Emeric CS, Magaziner JS, Adachi JD, Pieper CF, et al. (2007) Zoledronic acid and clinical fractures and mortality after hip fracture. N Engl J Med 357: 1799-1809.

56. Grey A, Bolland M, Wattie D, Horne A, Gamble G, et al. (2010) Prolonged antiresorptive activity of zoledronate: A randomized, controlled trial. J Bone Miner Res 25: 2251-2255.

57. Grey A, Bolland MJ, Horne A, Wattie D, House M, et al. (2012) Five years of anti-resorptive activity after a single dose of zoledronate - Results from a randomized double-blind placebo-controlled trial. Bone 50: 1389-1393.

58. Grey A, Bolland MJ, Wattie D, Horne A, Gamble G, et al. (2009) The antiresorptive effects of a single dose of zoledronate persist for two years: A randomized, placebo-controlled trial in osteopenic postmenopausal women. J Clin Endocrinol Metab 94: 538-544.

59. McClung M, Miller P, Recknor C, Mesenbrink P, Bucci-Rechtweg C, et al. (2009) Zoledronic acid for the prevention of bone loss in postmenopausal women with low bone mass: A randomized controlled trial. Obstet Gynecol 114: 999-1007.

60. Boonen S, Reginster JY, Kaufman JM, Lippuner K, Zanchetta J, et al. (2012) Fracture risk and zoledronic acid therapy in men with osteoporosis. N Engl J Med 367: 1714-1723.

61. Grey A, Bolland M, Wong S, Horne A, Gamble G, et al. (2012) Low-dose zoledronate in osteopenic postmenopausal women: A randomized controlled trial. J Clin Endocrinol Metab 97: 286-292.

62. Bai H, Jing D, Guo A, Yin S (2013) Randomized controlled trial of zoledronic acid for treatment of osteoporosis in women. J Int Med Res 41: 697-704.

63. Chao M, Hua Q, Yingfeng Z, Guang W, Shufeng S, et al. (2013) Study on the role of zoledronic acid in treatment of postmenopausal osteoporosis women. Pak J Med Sci 29: 1381-1384.

64. Dalbeth N, Aati O, Gamble GD, Horne A, House ME, et al. (2014) Zoledronate for prevention of bone erosion in tophaceous gout: a randomised, double-blind, placebo-controlled trial. Ann Rheum Dis 73: 1044-1051.

65. Barrett-Connor E, Swern AS, Hustad CM, Bone HG, Liberman UA, et al. (2012) Alendronate and atrial fibrillation: a meta-analysis of randomized placebo-controlled clinical trials. Osteoporos Int 23: 233-245.

66. Lewiecki EM, Cooper C, Thompson E, Hartl F, Mehta D, et al. (2010) Ibandronate does not increase risk of atrial fibrillation in analysis of pivotal clinical trials. Int J Clin Pract 64: 821-826.

67. Karam R, Camm J, McClung M (2007) Yearly zoledronic acid in postmenopausal osteoporosis. N Engl J Med 357: 712-713; author reply 714-715.
